# Progress in Medicine

**Published:** 1897-05-15

**Authors:** 


### Progress in Medicine.

### DISEASES OF THE DIGESTIVE ORGANS.

*(Continued from page 97.)*

**Pancreas.**—Hale White[1] discusses the signs of carcinoma. Out of 6,000 post-mortems there were nineteen cases of primary cancer of the pancreas, which was indeed the most common affection of that organ, apart from congestion or cirrhosis due to disease of the circulatory system. There were also sixteen instances of atrophy, chiefly diabetic, eight of fatty degeneration, four of cystic disease, and one of hæmorrhagic pancreatitis and three of calculi; besides other cases where secondary growths were found. Thus scirrhus carcinoma is the most important affection from a clinical point of view which we have to do with. It occurs most commonly in the head of the pancreas, and in some cases the symptoms may be surprisingly few. The most important are severe pain in the upper part of the abdomen, wasting, sometimes jaundice, or fatty stools when the secretions of the liver or the pancreas are blocked, and possibly a tumour to be felt on palpation. Constipation, or rarely diarrhœa, may occur, or the growth may ulcerate through the gastric or intestinal wall. The diagnosis may have to be made by exclusion, from the absence of symptoms due to disease of any of the neighbouring organs. In one of the two recent cases which he reports hæmatemesis appeared from ulceration through the wall of the stomach.

Chiari[2] considers that auto-digestion of the pancreas, as of the stomach, not infrequently takes place post-mortem, and even shortly before death.

Hæmorrhagic pancreatitis has been observed usually in persons of about forty-five years of age. McPhedran[3] has recently, however, recorded a unique case in a male infant of nine months. Vomiting and colic were followed by constipation, which did not yield to remedies. Intussusception was diagnosed on account of a sausage-like tumour, which turned out to be an accessory lobe of the liver. Post-mortem the middle third of the pancreas was found to be deeply infiltrated with blood. Newton Pitt and L. A. Parry[4] report another instance in a man of 60, who was seized with abdominal pain, vomiting, and absolute constipation. There was no collapse and little distension to indicate internal strangulation or volvulus, and when laparotomy was performed no obstruction could be found. The pancreas after death was infiltrated with blood, and patches of fatty necrosis marked the omentum in its neighbourhood, as well as the pancreas itself when stained. There was no peritonitis. Dr. Pitt remarks that when there is a tumour in the region of the pancreas more or less resonant, or evidence of a stone in the common duct, or of injury to the pancreas, together with the above symptoms, it is not difficult to diagnose the condition. The necrosed patches are, he thinks, due to extravasated pancreatic secretion. Another case occurred under the care of Mr. A. C. Hovenden,[5] of Surbiton, in August last. The patient was attacked with constipation, abdominal pain, and vomiting. Shortly before death, which occurred on the third day, the abdomen became distended, but there was no rise of temperature throughout. The pancreas was found much enlarged and of a uniform red colour. R. H. Kennan[6] relates yet another instance in a woman at Dublin, where death took place in 42 hours. The pancreas was intensely congested, and several gall-stones were present, one of which projected into the lumen of the duodenum. Some peritonitis had occurred, but no jaundice; nor was there a history of colic previously. A fifth case was described last year by Cayley.

Cysts in the pancreas have been ascribed to partial obstruction by calculi, to injury, and other causes. B. B. Morton[7] describes one in a man of 56 years, who had suffered for three months from pain in the gastric region, vomiting, and slight jaundice. A tumour was found in the left hypochondriac and gastric regions of the size of a fœtal head, unmoved by respiration. By aspiration a greenish-brown fluid was removed, alkaline in reaction, albuminous, and containing no bile pigment or sugar. A gall-stone was found, after death, loosely impacted in the common bile duct.

T. C. Railton[8] found a similar cyst in an infant of six months, which in this case extended from the ribs to the anterior superior spine of the ilium and from near the umbilicus quite round to the kidney. The case was regarded at first as one of hydronephrosis, from the lateral position of the cyst which sprang from the tail of the pancreas. There was no history of injury or of difficult birth.

**Spleen.**—Bland Sutton[9] discussed the subject of wandering spleens before the Medical Society of London recently. The mobility of these spleens is greater than that of any other organ, as they may travel to every corner of the peritoneal cavity and occasion endless difficulties in diagnosis. They are most often found in women who have borne children, and may become enormously enlarged and engorged with blood, especially when twisted, and in extreme cases of torsion atrophy or acute strangulation may occur. The other organs may be dragged out of place, and the uterus may be even retroflexed or prolapsed from the pressure. Thus, intestinal obstruction, rupture of the stomach intestine or spleen, or splenic abscess may occur. Sutton finds that splenectomy is not only justifiable but highly successful. Ridygier has apparently succeeded, however, in carrying out a promising method of fastening the spleen in its place. After splenectomy the patients seem to recover normal health, but remain always thin. M. Balance noticed in two cases, after the operation, pyrexia, anæmia, leucocytosis, and great emaciation. Ultimately the external lymphatic glands enlarged and the patients recovered. In young animals the red marrow takes up the functions of the spleen, and the lymphatic glands those of the malpighian bodies after splenectomy. Sutton believes that the symptoms referred to by Balance are quite exceptional.

**Peritoneum.**—Schramm[10] met with a case of chylous ascites in a woman aged 53, who had wasted away for six months. The abdomen was distended with no less than 16 litres of milky fluid. Death resulted from peritonitis, and malignant growths were found in many parts of the body. The thoracic duct was invaded and obliterated by the growth, but it was uncertain where this had commenced, except that the liver and gall-bladder were not the starting-point, though there were 20 stones in the gall-bladder. In five other recorded cases of carcinoma of the duct no chylous ascites occurred. Hence the duct could not have been obliterated, or else collateral channels were opened. In this patient there was complete obstruction, and the fluid seemed to transude through the altered walls, since there was no rupture of the duct discoverable.

Codd, of Liverpool, reports another case in the *British Medical Journal* of May 1st. The patient suffered from mitral disease and bronchitis. The external and internal jugular and other veins were blocked by thrombosis, and the thoracic duct was dilated by the obstruction. Four and a half pints of chylous fluid were found in the peritoneum.

Byron Robinson[11] has investigated the effects of adhesions on the digestive tract in three hundred autopsies. They occur usually from the friction of the overlying muscles and microbic invasion. The latter may gain entrance through the Fallopian tubes, or through the coats of the bowel when irritated by muscular action, such as that of the psoas and diaphragm. These adhesions may cause much pain and dyspepsia when they check peristalsis or fix mobile viscera, but rarely, if ever, when they affect more fixed organs, as the cæcum, liver, or spleen. Most serious of all is their action when a peritonitic band is fixed to the most mobile point of an organ, such as the middle of the sigmoid, causing it to form an acute angle. In order of frequency these local attacks of peritonitis are found most often round the spleen, then about the meso-sigmoid, in the region of the appendix, next in that of flexure of the colon, the Fallopian region, the meeting-place of the duodenum and crus of the diaphragm, and a spot on the opposite crus behind the stomach.

G. R. Fowler,[12] in discussing the dangers of appendicitis, lays down that mild recurrent attacks may pass off harmlessly unless they result in causing obliteration of the canal at one or more points, and quotes a case where previous occlusion left a cavity in which an ulcer started when fresh irritation took place. If entire obliteration of the canal takes place there is little danger. Sivedey and Le Roy[13] notice that the "closed cavity" theory of appendicitis does not account for all cases, and that the fundamental lesion is severe inflammation of the follicles of the deeper parts of the mucosa, but simultaneous changes may be found above or below this region.

Herniæ are frequently the real cause of dyspeptic symptoms, which disappear on treatment of the hernia. Schütz[14] finds the small ones of the linea alba are often overlooked, and cause many symptoms such as colicky pains, constipation, and flatulence. They are seen most often above the umbilicus and to the right of the median line, and occasionally in the mammillary line.

**Intestines.**—Simple perforating ulcers are usually said to be limited to the stomach, duodenum, and lower part of the œsophagus; but F. O. Simpson[15] reports one which was found in the jejunum, six inches below its commencement. The patient was indeed a half-witted melancholic, and may have eaten some substance which acted as a foreign body. The co-existence of protozoan and bacterial infections is rare. Quite occasionally malaria is found together with amœbic dysentery. Simon Flexner[16] describes a case of diarrhœa with ulceration, appendicitis, and small liver abscesses, in which the amœba, as well as Fränkel's diplococcus was present. It is claimed that a dessertspoonful of peeled garlic boiled in milk and given every two hours is a specific for dysentery. A. P. Pillay[17] adds to this for chronic cases a drachm of powdered cinnamon, with half a drachm of cloves and a quarter of nutmeg, and administers the mixture three times a day. We owe a valuable paper on dysentery to the pen of J. Maberley,[18] who had much experience in South Africa. He advocates a new remedy, the tincture of monsonia, as effective both in acute and chronic cases. Since the revival of Docker's Ipecacuanha treatment the mortality of acute attacks in India is said to have been reduced to less than five per cent., but chronic cases are very intractable. Maberley made an ordinary tincture from dried leaves and stems of the monsonia ovata, a native plant, which he found invaluable in two or four drachm doses every four hours. He reports the results in 100 cases; ten of these were chronic, nine of which recovered completely with an average duration of treatment of eight days. The average treatment in all his cases was two and a half days. Large injections of Condy or soap and water were also useful to relieve the small intestine which ceases to pass on fæcal matter when the disease is established. In the absence of the tincture of monsonia the author found phenacetin of value as an addition to the usual treatment. Testevin[19] treats dysentery by hypodermics of morphine, one-twelfth of a grain every hour, mustard poultices or ice-bags, and repeated small doses of calomel with ipecacuanha and opium for two or three days, as well as rectal injections of creasote, and borax solutions. The treatment of intestinal dyspepsia is frequently most difficult. H. S. Cumming[20] finds taka diastase of great value if the diet and gastric digestion are looked after. Armstrong, of Buxton,[21] also recommends it in doses of three to five grains with the meals in cases of amylaceous or gouty dyspepsia. Where intestinal catarrh exists, Upshur[22] recommends 30 grains of phosphate of soda every two hours in hot water. Guntzburg has used with much success icthyol pills (coated with keratin to avoid eructations), to relieve both diarrhœa and constipation, especially in women suffering from genito-urinary disorders. Sahli[23] found that gelatine capsules hardened by formaldehyde completely resist gastric digestion, and can, therefore, be employed for conveying active drugs to the intestines. An account of the literature of tannigen and tanalbin is given in the *Medical Chronicle* of November. Both pass through the stomach in an inert form, and liberate tannin in the intestine. Müller gave 10 to 60 grains of tannigen daily, with success, in chronic diarrhœa, even that of phthisis, but thinks it of little use in the diarrhœa of children. Künkler, Drews, and De Buck, however, speak highly of it in these cases, and notice that it must not be given in milk. Tanalbin has been used by Vierordt and Von Engel in larger doses, half a drachm and upwards several times a day, and both speak highly of it. Mucous colitis is regarded as a nervous affection by W. Mendelson,[24] like the salivation and polyuria of hysteria. It is remarkable for its very long duration, and the severe pain which accompanies the passage of the mucous casts in the morning stools. He treats it by very large injections of boric acid solutions, rest in bed, and highly nourishing diet, telling his patients that if they gain 10 pounds in weight they will be cured.

[1] Lancet, Dec. 26. [2] Amer. J. Med. Sc., Dec. [3] Lancet, Jan. 9. [4] Lancet, Jan. 2. [5] Lancet, Jan. 2. [6] B. Med. Jour., Nov. 14. [7] Lancet, Jan. 23. [8] B. Med. Jour., Oct. 31. [9] B. Med. Jour., Jan. 16. [10] Amer. Jour. Med. Sc., Jan. [11] Med. Rec., Nov. 28. [12] Med. Rec., Nov. 7. [13] Med. Week, Feb. 5. [14] Med. Rec., Nov. 28. [15] Practitioner, Feb. [16] Johns Hopkins Bul., 66, 67, Vol. VII. [17] Amer. Jour. Med. Sc., Dec. [18] Lancet, Feb. 6. [19] Therap. Gazette, Dec. [20] Therap. Gazette, Oct. [21] Lancet, March 13. [22] Therap. Gazette, Dec. [23] Deutsch. Med. Woch, Jan. 1. [24] Med. Rec., Jan. 30.

